# New pulse wave measurement method using different hold-down wrist pressures according to individual patient characteristics

**DOI:** 10.1186/2193-1801-2-406

**Published:** 2013-08-27

**Authors:** Seong Ki Yoo, Ki Young Shin, Tae Bum Lee, Seung Oh Jin

**Affiliations:** Advanced Medical Device Research, Korea Electrotechnology Research Institute, 111 Hangaul-ro, Sangnok-gu, Ansan, Gyeonggi-do 426-910 Korea

**Keywords:** Pulse wave measurement, Pulse depth, Skin thickness, Floating/Sinking pulse pattern, Hold-down pressure

## Abstract

In traditional Chinese and Korean medicine, doctors first observe a patient’s pulse by gently and strongly pressing their fingers onto the wrist, and then make a diagnosis based on the observed pulse waves. The most common method to implement this diagnostic technique is to mechanically extract the pulse waves by applying a fixed range of pressures for all patients. However, this method does not consider the patients individual characteristics such as age, sex, and skin thickness. In the present study, we propose a new method of pulse wave extraction that incorporates the personal characteristics of the patients. This method measures the pulse wave signal at varying hold-down pressures, rather than applying a fixed hold-down pressure for all patients. To compare this new technique with existing methods, we extracted pulse waves from 20 subjects, and then determined the actual applied pressure at each step, the coefficient of floating and sinking pulse (CFS), and the distinction of floating/sinking pulse for each group. Consequently, each subject had a different pressure range in our proposed method, whereas all subjects had a similar pressure range in the existing method. Four of 20 subjects exhibited different floating/sinking pulse patterns due to the value of the first pressure step and the range of hold-down pressures. These four subjects were categorized as overweight based on BMI. In addition, the moving distance of the proposed method was longer than the existing method (p = 0.003, paired t-test), and the correlation coefficient between CFS values of two different methods was 0.321, indicating that there was no correlation.

## Background

In Traditional Chinese Medicine (TCM) and Korean Medicine (KM), four diagnostic methods of observation (listening, smelling, inquiring, and palpation) are used to diagnose diseases in patients. Pulse diagnosis is the representative diagnostic method belonging to the palpation diagnostic methods (Kim and Kang 2008). The purpose of pulse diagnosis is to determine evolution of a disease, causes of a disease, position of a disease, and a cure for the disease. Pulse diagnosis is traditional and venerable. However, it is difficult to become proficient in measurement of pulse waves, and the measurement and analysis of pulse waves is subjective. Therefore, a pulse diagnosis can vary with different doctors. A number of studies have attempted to objectify and quantify pulse waves to overcome this problem (Ryu et al. 2007; Kim et al. 1999) by using pulse diagnosis sensors, pulse wave simulators, and pulse diagnosis instruments (or pulse taking devices) (Fu and Lai 1989 Jeon et al. 2008; Kim et al. 2009a; Luo et al. 2012; Shin et al. 2010; Shin et al. 2011; Yoo et al. 2013). A pulse diagnosis sensor has been developed for pressure calibration, size, temperature and deployment, while a pulse wave simulator has been developed for an objective standard for pulse analysis. These advancements mainly focus on development of hardware to acquire objective and quantitative pulse waves. In the present study, we developed a software-based pulse wave measurement method.

The pulse diagnosis in KM has 28 representative pulse patterns determined by pulse parameters such as rate, rhythm, arterial width, depth, length, arterial tension, force, ease of occlusion, and pulse contour. Ten of 28 pulse patterns have particularly high clinical utility, and include floating/sinking pulse (defined by level of depth), slow/rapid pulse (defined by rate), long/short pulse (defined by length), vacuous/replete pulse (defined by force), and broad/fine pulse (defined by width). The floating/sinking pulse is associated with the hold-down pressure that can vary with individual patients. For example, a slim person’s radial artery may be more easily accessible because of the thinner layer of subcutaneous tissue, while someone with a higher proportion of body fat may have an artery that is more difficult to palpate because of the thicker subcutaneous tissue layer. As such, the level of hold-down pressure may differ from person to person. TCM and KM doctors distinguish between floating pulse and sinking pulse by the difference of pulse pressure felt at different levels of hold-down pressure. To implement applying weak and strong pressure on wrist by a mechanical system, existing pulse wave measurement methods apply a constant range of pressure for all subjects. Most pulse diagnosis instruments in common use have adopted this method. A 3D MAC (Daeyomedi, Korea) is a representative pulse diagnosis instrument. This instrument applies five different levels of hold-down pressure to the wrist, and measures pulse wave signals by using a piezoresistive pressure sensor and a control robot. A P-H curve is then generated by spline interpolation, and the pulse wave is analyzed from the measured pulse wave signals at five pressure steps. The concept of a P-H curve with hold-down pressure on the horizontal axis and pulse wave pressure on the vertical axis was introduced in KM. In addition, the distinction of pulse patterns such as floating/sinking pulse and rapid/slow pulse and diagnosis of chronic gastritis have been studied by using pulse waves measured by this method (Kim et al. 2009b; Kim and Shin 2010; Shin et al. 2012). The existing method has the advantage of measuring pulse wave signals stably at each of five pressure steps. However, it does not consider that the hold-down pressure applied by an oriental doctor varies from person to person, as the sensitivity of the doctor’s finger varies with personal characteristics such as age, sex, and skin thickness. Therefore, the existing method of applying the same value of hold-down pressure can be problematic for acquisition and analysis of pulse wave signals (Barker 1951; Lee et al. 2008).

Therefore, in this study we propose a new method for measuring pulse wave signals that considers the personal characteristics of the patients. The proposed method measures pulse wave signal at varying values of hold-down pressure for each subject. To achieve this, the pulse diagnosis instrument constantly applies increasing pressure and measures the pulse waves until they reach the termination point. The start and last points are then detected, and are used to determine the specific pressure range in which the sensor detects the subjects pulse waves. The range is then equally divided into five steps, and at each step the subjects pulse waves are finally extracted and analyzed. We compared the measurements of floating/sinking pulse, pulse depth (motor moving distance), and actual applied pressure (AAP) at each of the five pressure steps between the two methods. The coefficient of floating and sinking pulse (CFS) is used to distinguish floating/sinking pulse (Lee et al. 2005).

## Methods

### Existing measurement methods

**Figure 1:**
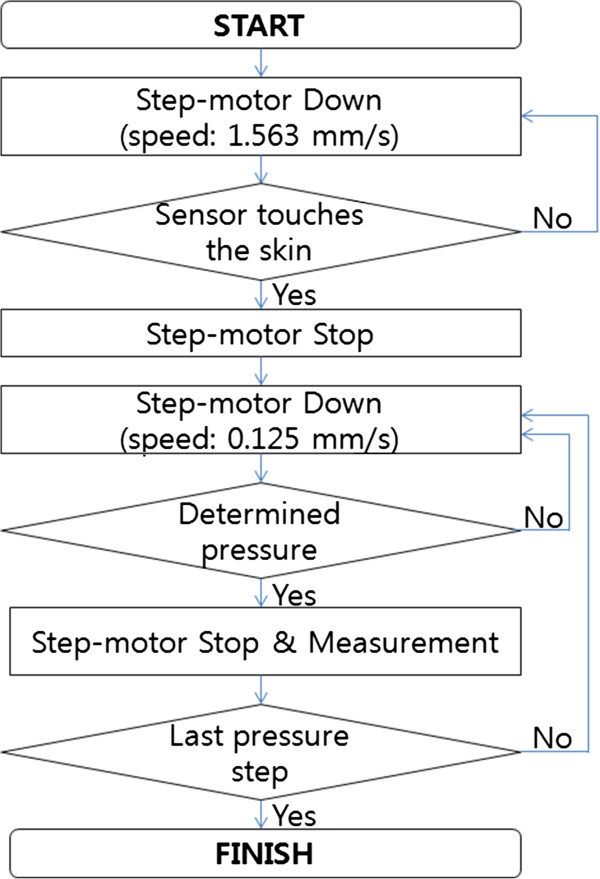
**Flow chart of the existing method.**

Current methods used to measure pulse waves involve applying a fixed range of pressures, regardless of the characteristics of the subject. The range of hold-down pressures and speed of the motor are determined by the manufacturer, and can differ from instrument to instrument. In this study, pressure was applied from 40 to 240 mmHg at 50-mmHg intervals (Figure 1). First, the pulse diagnosis instrument moves a pulse diagnosis sensor down at 1.563 m/s (fast) by a step-motor. When the pulse diagnosis sensor touches the skin, the step-motor immediately stops moving. Next, the sensor moves down at 0.125 m/s (slow), this time to detect hold-down pressure precisely. Once the hold-down pressure reaches the determined pressure for each step (1^st^ pressure step: 40 mmHg; 2^nd^ pressure step: 90 mmHg; 3^rd^ pressure step: 140 mmHg; 4^th^ pressure step: 190 mmHg; Last pressure step: 240 mmHg), the pulse diagnosis sensor stops and measures the pulse waves. This process is repeated until the hold-down pressure reaches the last pressure step.

### Proposed method

**Figure 2:**
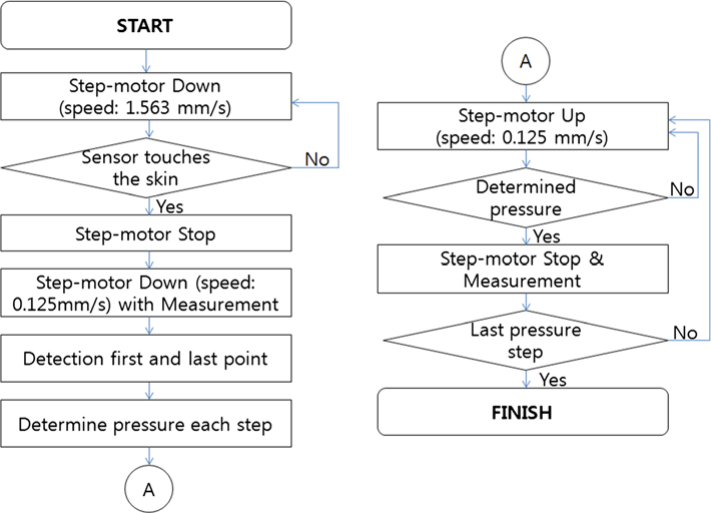
**Flow chart of the proposed method.**

**Figure 3:**
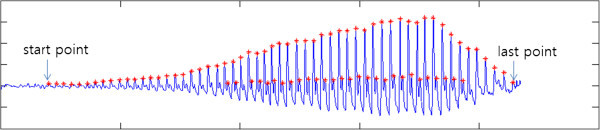
**Detection of the first and last pulse points.**

The proposed method measures the pulse waves at five different pressure steps over a unique range determined for each patient. Therefore, the value of the hold-down pressure at each step is not fixed. A flow chart shown in Figure 2 describes the operation of the proposed method. First, a pulse diagnosis instrument moves a pulse diagnosis sensor down at 1.563 m/s (fast) by a step-motor. When the pulse diagnosis sensor touches the skin, step-motor immediately stops moving. The sensor then moves down at 0.125 m/s (slow) until a pulse is not perceived, measuring pulse waves continuously. From the measured pulse wave, local maximum values (red asterisk) can be found by the peak detection method. As shown in Figure 3, the first local maximum value and last local maximum value are determined as the two points where the pulse is sensed first and last, respectively. The hold-down pressure at the two points is then determined and divided into five steps. For example, if the hold-down pressures at the first and last points are 25 mmHg and 245 mmHg, respectively, the hold-down pressure at each step is determined as 1^st^ pressure step: 25 mmHg, 2^nd^ pressure step: 80 mmHg, 3^rd^ pressure step: 135 mmHg, 4^th^ pressure step: 190 mmHg, and 5^th^ pressure step: 245 mmHg.

The sensor then moves up at 0.125 m/s (slow). Once the hold-down pressure reaches the determined pressure for each step as listed above, the pulse diagnosis sensor stops and measures the pulse waves. This process is repeated until the hold-down pressure reaches the first pressure step. Note that motor down denotes increased hold-down pressure, while motor up denotes decreased hold-down pressure.

### Comparison of the two methods

**Figure 4:**
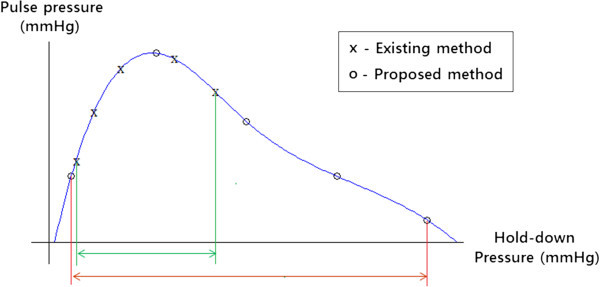
**Comparison of hold-down pressure range between the two methods.**

The main difference between the existing method and the proposed method is the range of hold-down pressures. The existing method measures pulse wave in a fixed hold-down pressure range, while the proposed method obtains the pulse wave over a hold-down pressure range that is defined according to the characteristics of the patient (i.e., the hold-down pressure at each step is different). As shown in Figure 4, the pulse wave signal at an inappropriate depth will be measured if the fixed range of hold-down pressure is not suitable for a particular subject. Figure 4 shows that the proposed method measures pulse wave at the proper depth, while the existing method obtains a pulse wave at an inappropriate depth. Therefore, it is important to apply appropriate pressure to acquire accurate pulse wave information.

### Experiments

**Figure 5:**
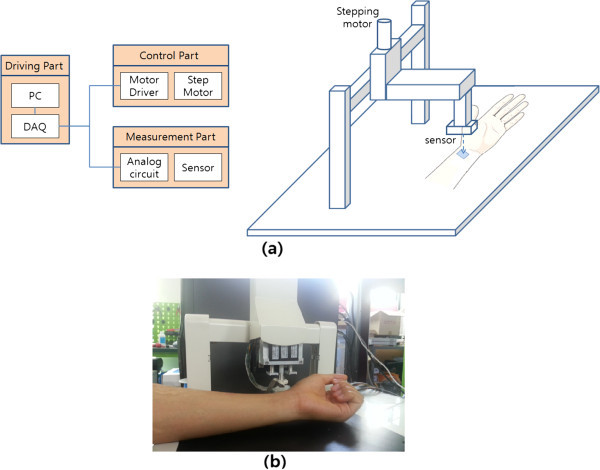
**Pulse diagnosis instrument. (a)** Block diagram; **(b)** Actual appearance.

The block diagram and actual appearance of the pulse diagnosis instrument are shown in Figure 5. The instrument consists of driving, measurement, and control parts. The driving part is composed of a step-motor (Motorbank, NK215-01AT, Korea) and a motor-drive (Jeilmotor, JUD203S16, Korea). It applies pressure onto the subject’s wrist by moving the pulse diagnosis sensor up and down. The measurement part is composed of a pulse diagnosis sensor and analog circuits. The pulse diagnosis sensor consists of six piezoresistive pressure sensors and one Negative Temperature Coefficient (NTC) thermistor. This piezoresistive pressure sensor generates mV output according to the applied pressure. This output is amplified and filtered by analog circuits. A NTC thermistor monitors the temperature change and compensates for the output change of the pressure sensors according to the measured temperature. The control part is composed of a Data Acquisition (DAQ) board (NI USB-6210; National Instruments Co., Austin, TX, USA) and a PC. The controlling part controls the driving part and monitor and saves the outputs of the measuring part.

**Figure 6:**
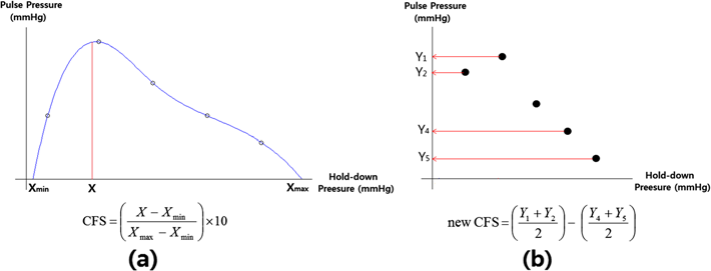
**Illustration and computation of the CFS. (a)** CFS (Lee et al. 2005); **(b)** New CFS (Kim et al. 2009b).

The pulse diagnosis instrument was employed to measure the pulse waves of 20 subjects using the two different methods. These pulse waves were collected at a sampling rate of 500 Hz for 10 s at each of the five pressure steps. Of the subjects, there were 15 males and 5 females. The average age, height, and weight of the 20 subjects were 32 ± 5.29 years, 173 ± 6.35 cm, and 69.25 ± 9.23 kg, respectively. Informed consent was obtained from all subjects before pulse signal measurement and the same investigator, who holds a medical laboratory technologist license, obtained all measurements. The number of pulses of the step-motor was collected to calculate the step-motor moving distance from the first pressure step and the last pressure step. The CFS and APP at each of the five pressure steps, and the pulse depth, were then calculated using the measure pulse waves and pulses to compare the two different methods. The pulse depth denotes the motor moving distance from the first pressure step to the last pressure step. To compare the moving distance of the two methods, a paired t-test was performed with a significance level of 0.05. The CFS was calculated by using Equations (1) and (2).1

where *X*_*min*_ is the minimum hold-down pressure, *X*_*max*_ is the maximum hold-down pressure, and *X* is the hold-down pressure where the maximum pulse pressure appears (see Figure 6a).2

where, *Y*_*n*_ is the pulse pressure at the n^th^ pressure step. This considers the average of the pulse pressures at the first and second pressure step as weak pressure, while the pulse pressures at the 4^th^ and 5^th^ pressure step are considered the strong pressure (see Figure 6b). The distinction of the floating/sinking pulse is defined according to CFS. In addition, a correlation analysis between the CFS values of two different methods was performed.

## Results

**Figure 7:**
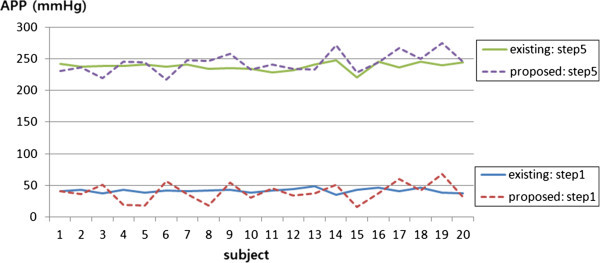
**APP at the first pressure step and the last pressure step of 20 subjects.**

Figure 7 shows APP at the first pressure step and the last pressure step of 20 subjects. The minimum and maximum values of the APP were 35 mmHg and 247 mmHg, respectively, in the existing method, and 16 mmHg and 278 mmHg, respectively, using our new method. In the existing method, APP at the first step and last step were similar to the determined value at the first and fifth pressure step. Conversely, the proposed method was able to measure the pulse wave signal at lower and greater pressure than the first and fifth steps of the existing method, as the proposed method detects the start point and last point of the pulse wave and divides this into five pressure steps. In the existing method, the mean value of actual pressure at each step was 41.75 ± 3.3 mmHg for step 1, 91 ± 4.5 mmHg for step 2, 141.5 ± 5.6 mmHg for step 3, 190.25 ± 6.3 mmHg for step 4, and 237.8 ± 6.2 mmHg for step 5. By contrast, in the proposed method the mean value of actual pressure at each step was 39.2 ± 14.7 mmHg for step 1, 87.4 ± 9.3 mmHg for step 2, 138.35 ± 7.9 mmHg for step 3, 189.25 ± 9.1 mmHg for step 4, and 243.25 ± 15.6 mmHg for step 5. Although these average actual pressures at each step were similar between the two methods, the standard deviations (SD) at each step were obviously different, suggesting that the proposed method applies different hold-down pressure and measures pulse wave signals according to personal characteristics.

Table 1 shows the results of the existing and the proposed methods, including value of CFS, distinction of floating/sinking pulse, and motor moving distance from the first pressure step and the last pressure step of the 20 subjects. The correlation coefficient between CFS values of two different methods was 0.321, implying that they were not significantly correlated. Four out of twenty subjects exhibited different results in the distinction of floating/sinking pulse pattern (subject 4, 5, 8, and 15). These four subjects were categorized as overweight by body mass index (BMI), while the remaining subjects were categorized as normal weight. The existing method indicates that these four subjects have a sinking pulse, whereas the proposed method shows that they have a floating pulse. The hold-down pressures data showed that the value of first pressure step in the proposed method was approximately 24 mmHg lower than that with the existing method. In addition, the range of hold-down pressures in the proposed method was approximately 28 mmHg wider than that using the existing method. The mean value of motor moving distance was 3.375 ± 0.994 mm in the existing method and 4.089 ± 1.183 mm in the proposed method (p < 0.001).

**Table 1:** **Results of the existing method and proposed method with respect to pulse depth**

Subject	BMI	Step1 - Step 5 moving distance (mm)		CFS		Distinction of floating/sinking pulse pattern		New CFS	
		Existing	Proposed	Existing	Proposed	Existing	Proposed	Existing	Proposed
1	21.14	2.85	2.64	4.384492	3.385465	Floating	Floating	0.594	8.949
2	21.56	5.91	6.23	5.139147	5.059429	Sinking	Sinking	7.470	11.752
3	20.89	3.42	2.97	5.031552	6.724444	Sinking	Sinking	−3.896	−9.181
4	**26.92**	**2.95**	**3.26**	**6.527585**	**3.335057**	**Sinking**	**Floating**	−5.381	11.946
5	**27.77**	**3.43**	**4.04**	**5.087282**	**2.153485**	**Sinking**	**Floating**	−3.156	11.002
6	23.51	2.39	1.87	7.011686	6.520645	Sinking	Sinking	−14.059	−7.031
7	18.73	4.99	5.06	5.068592	6.613833	Sinking	Sinking	−3.7858	−6.58355
8	**30.24**	**2.89**	**4.04**	**7.038385**	**2.393842**	**Sinking**	**Floating**	−3.5957	5.1531
9	23.67	4.79	4.82	2.851511	2.613861	Floating	Floating	5.78025	3.71175
10	23.29	2.72	5.85	5.061054	7.596154	Sinking	Sinking	−5.8969	−15.498
11	20.42	2.97	4.11	2.382716	4.474097	Floating	Floating	6.0137	3.92475
12	20.95	4.22	5.46	4.998414	4.072165	Floating	Floating	−1.0088	−2.8427
13	19.13	2.36	3.8	3.193309	4.607309	Floating	Floating	8.77415	0.74365
14	22.98	4.5	5.53	4.331481	2.130134	Floating	Floating	12.6802	9.302215
15	**26.77**	**3.3**	**4.66**	**5.802519**	**2.177047**	**Sinking**	**Floating**	−3.4977	4.98385
16	21.71	2.92	4.04	3.184143	2.743239	Floating	Floating	4.22665	3.0999
17	22.47	3.08	2.53	2.643532	1.933152	Floating	Floating	5.53729	4.3691
18	23.72	3.12	4.23	2.538462	3.013174	Floating	Floating	3.32625	5.9928
19	21.25	2.33	2.83	3.143128	2.408015	Floating	Floating	6.7572	3.1933
20	20.91	2.36	3.81	2.047177	2.329227	Floating	Floating	12.2276	11.17855
Mean	-	**3.375**	**4.089**	-	-	-	-	-	-
S.D	-	0.994	1.183	-	-	-	-	-	-

## Discussion

In this study, we developed a new pulse wave measurement method that adjusts for the personal characteristics of the patients including age, sex, and skin thickness. We compared the pulse waves of 20 subjects between existing and our proposed methods. The APP at each of the five pressure steps and moving distance between the first pressure step and the last pressure step were calculated. In the existing method, the APP of 20 subjects at each of the five pressure steps were similar, as the range of the pressures applied onto each subjects’ wrist was the same. However, in the proposed method the APP of 20 subjects at each of the five pressure step was different because the proposed method measures the pulse wave signal at a hold-down pressure that varies according to individual subject characteristics. The result of the proposed method shows that pulse waves can be measured at pressures between 40 and 240 mmHg. In addition, the step-motor moving distance in the proposed method is longer than that in the existing method, suggesting that the proposed method perceives and measures pulse wave at deeper a level, while the existing method might miss pulse wave information at a deeper level and therefore provide inaccurate depth data. Although the range of hold-down pressures determined using the existing method in our study was also from 40 to 240 mmHg, the majority of existing pulse diagnosis instruments have a smaller range, and are more likely to provide inaccurate depth data. Therefore, the existing method is not suitable for subjects who have a pulse signal beyond the range of hold-down pressures. Conversely, the proposed method minimizes the overlooked pulse wave signal by varying the hold-down pressure from person to person.

In the present study, we calculated the value of CFS to distinguish between floating/sinking pulses. We found that four subjects who were categorized as overweight based on BMI exhibited different floating/sinking pulse patterns, while the remaining normal weight subjects exhibited the same pattern. Therefore, the thicker subcutaneous tissue layer of overweight subjects exerts an influence on pulse wave measurement. On closer examination, the existing method indicated that these four subjects had a sinking pulse, whereas the proposed method showed a floating pulse. This difference may have occurred when the hold-down pressure was strong enough to perceive pulse signal at a deep level. Although a new CFS value was calculated in the present study, it was not used to distinguish between floating/sinking pulses, as this distinction can only be defined when all pulse waves on Chon, Kwan, and Chuck position are measured (we measured the pulse wave only on Kwan). Nevertheless, we determined that the correlation coefficient between CFS values for the two different methods was 0.505, indicating that they were not significantly correlated.

### Summary

Our proposed method could successfully measure pulse waves accounting for individual subjects characteristics. However, although the correlation analysis and pair t-test indicate a difference between the methods, we were unable to conclude which method is dominant. Therefore, further studies are required to compare CFS results of the two different methods with analysis of floating/sinking pulse by professional TCM and KM doctors, and by measuring hold-down pressure when a TCM/KM doctor palpates with a different level of hold-down pressure.
